# *In vivo* assessment of the recovery of myocardial pyruvate dehydrogenase activity following a ketogenic diet

**DOI:** 10.1093/cvr/cvag053

**Published:** 2026-02-26

**Authors:** Jun Chen, Zohreh Erfani, Abdallah Elnwasany, Sarah Al Nemri, Joseph Park, Mai T Huynh, Maheen Zaidi, Crystal E Harrison, Xiaodong Wen, Luke I Szweda, Jae Mo Park

**Affiliations:** Advanced Imaging Research Center, UT Southwestern Medical Center, 5323 Harry Hines Blvd. Dallas, TX 75390, USA; Advanced Imaging Research Center, UT Southwestern Medical Center, 5323 Harry Hines Blvd. Dallas, TX 75390, USA; Division of Cardiology, Department of Internal Medicine, UT Southwestern Medical Center, Dallas, TX 75390, USA; Department of Biology, University of Dallas, Irving, TX 75062, USA; Advanced Imaging Research Center, UT Southwestern Medical Center, 5323 Harry Hines Blvd. Dallas, TX 75390, USA; Advanced Imaging Research Center, UT Southwestern Medical Center, 5323 Harry Hines Blvd. Dallas, TX 75390, USA; Advanced Imaging Research Center, UT Southwestern Medical Center, 5323 Harry Hines Blvd. Dallas, TX 75390, USA; Advanced Imaging Research Center, UT Southwestern Medical Center, 5323 Harry Hines Blvd. Dallas, TX 75390, USA; Advanced Imaging Research Center, UT Southwestern Medical Center, 5323 Harry Hines Blvd. Dallas, TX 75390, USA; Advanced Imaging Research Center, UT Southwestern Medical Center, 5323 Harry Hines Blvd. Dallas, TX 75390, USA; Division of Cardiology, Department of Internal Medicine, UT Southwestern Medical Center, Dallas, TX 75390, USA; Department of Medicine, University of Arizona, Tucson, AZ 85719, USA; Advanced Imaging Research Center, UT Southwestern Medical Center, 5323 Harry Hines Blvd. Dallas, TX 75390, USA; Department of Biomedical Engineering, UT Southwestern Medical Center, 5323 Harry Hines Blvd, Dallas, TX 75390, USA; Department of Radiology, UT Southwestern Medical Center, 5323 Harry Hines Blvd, Dallas, TX 75390, USA; Charles and Jane Pak Center for Mineral Metabolism and Clinical Research, UT Southwestern Medical Center, 5323 Harry Hines Blvd, Dallas, TX 75390, USA

**Keywords:** Ketogenic diet, Hyperpolarization, Pyruvate dehydrogenase, Cardiac metabolism, Metabolic flexibility

## Abstract

**Aims:**

A ketogenic diet (KD) can suppress cardiac carbohydrate utilization, which may adversely impact heart function. However, the reversibility of KD-induced metabolic changes is poorly understood. This study aims to characterize myocardial pyruvate dehydrogenase (PDH) flux during the transition from a prolonged KD to a normal chow diet (ND).

**Methods and results:**

Cardiac metabolism was longitudinally assessed in rats using hyperpolarized [1-^13^C]pyruvate at baseline, during a KD (2 and 5 weeks), and a subsequent ND (1, 2, 5, and 8 days) after the 5-week KD. Hyperpolarized ^13^C products were compared between the KD group and age-matched ND controls. In parallel, nuclear magnetic resonance isotopomer analysis of cardiac tissue with an injection of [3-^13^C]pyruvate and [1,2-^13^C_2_]acetate was performed along with *ex vivo* enzymatic analysis of PDH activity. Myocardial [^13^C]bicarbonate production relative to total ^13^C products decreased from 8.56 ± 2.29% at baseline to 0.46 ± 0.27% after 5 weeks of KD. Reverting to ND gradually restored PDH flux (8.40 ± 1.47% by Day 8) to control levels (8.69 ± 2.10%). *Ex vivo* NMR analysis of glutamate C4 showed reduced pyruvate contribution to acetyl-CoA during KD (4.1 ± 2.5%), which recovered upon reverting to ND (22.7 ± 1.82% vs. control: 27.6 ± 9.5%). Although PDK4 expression normalized, PDH activity remained partially impaired in the reverted group (36.80 ± 6.07 mmol NADH/min/mg) compared to controls (90.97 ± 5.40; *P* = 0.00007).

**Conclusion:**

KD-induced suppression of myocardial PDH flux is reversible, but its recovery requires significant time, with prolonged metabolic inflexibility persisting after transitioning to an ND. These findings highlight the value of *in vivo* assessment of cardiac PDH activity, complemented by conventional enzymatic analyses, to identify persistent metabolic inflexibility following ketogenic interventions.

## Graphical Abstract

**Graphical Abstract:**
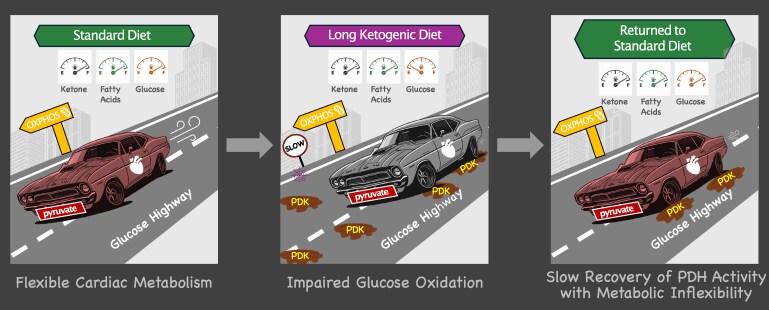

**Time of primary review: 29 days**

## 1. Introduction

Fatty acids and glucose are principal substrates for cardiac metabolism to support adenosine triphosphates (ATP) production. Fractional contributions can rapidly adapt to substrate availability, workload, circadian rhythm, pathological conditions, and nutritional states.^1^ Glucose metabolism plays a key role in providing the metabolic flexibility, which is strongly linked to cardiac damage and dysfunction.^2^ For instance, the utilization of fatty acids increases in the heart under a fasted condition due to limited glucose availability. The diminished glucose oxidation is, in part, driven by increased expression of pyruvate dehydrogenase kinase 4 (PDK4) and phosphorylation and inhibition of pyruvate dehydrogenase (PDH).^3,4^ Since PDH is the rate limiting step in the oxidation of glucose-derived pyruvate for ATP production within the mitochondria, measuring the PDH flux is essential for evaluating cardiac glucose metabolism.

**Figure 1:**
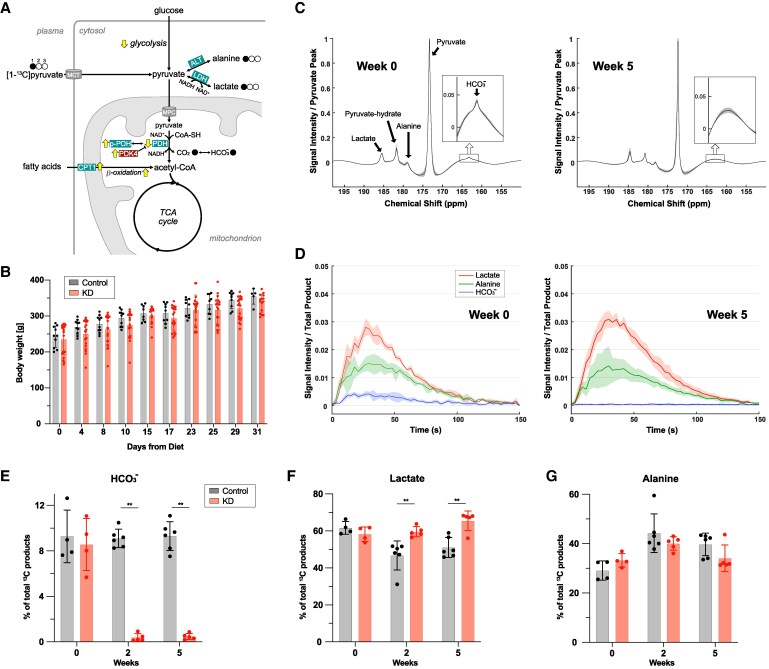
Longitudinal monitoring of skewed cardiac pyruvate metabolism by KD using HP [1-^13^C]pyruvate. (*A*) Cardiac metabolism altered by KD and the pathway of labelled carbon when HP [1-^13^C]pyruvate is injected. The solid circle and empty circle represent labelled (^13^C) and unlabelled (^12^C) carbons, respectively. The yellow arrow indicates metabolic changes induced by a KD. (*B*) Body weights of the rats during a KD and a ND. (*C*) Accumulative ^13^C spectra averaged from the KD group at baseline and after 5 weeks of KD. (*D*) Time courses of HP [1-^13^C]lactate, [1-^13^C]alanine, and H^13^CO_3_^−^ at baseline and after 5 weeks of KD. The shaded spectra in (*C*) and shaded time-courses in (*D*) indicate +/− standard deviation. Changes of relative metabolite levels to total HP products from the CT (*n* = 6) and KD groups (*n* = 5): (*E*) H^13^CO_3_^−^/TP, (*F*) [1-^13^C]lactate/TP, and (*G*) [1-^13^C]pyruvate/TP. Two-way ANOVA tests were used (α = 0.05). ** indicates *P* < 0.01. TP, total HP ^13^C products; KD, ketogenic diet; ND, normal diet.

Considering the sensitive enzymatic activities to multiple physiological factors and complex regulatory pathways, glucose oxidation in the heart needs to be understood *in vivo*. In particular, the PDH activity is sensitive to various factors such as plasma substrate composition and insulin level,^5,6^ it is more reliable to measure the activity *in vivo*. ^13^C magnetic resonance spectroscopy (MRS) with a bolus injection of hyperpolarized (HP) [1-^13^C]pyruvate enables *in vivo* assessment of PDH flux via the detection of [^13^C]bicarbonate (H^13^CO_3_^−^), which is formed from ^13^CO_2_ generated during the conversion from [1-^13^C]pyruvate to acetyl-CoA (*Figure 1, A*).^7,8^ One of the most exciting motivations of cardiac MRI/MRS with HP [1-^13^C]pyruvate is its capability of assessing PDH flux *in vivo* with systemic factors present. The utility of HP H^13^CO_3_^−^as a non-invasive biomarker of *in vivo* PDH activity has been demonstrated in both preclinical studies under various nutritional and pathological conditions^9–12^ and translational studies with patients.^13–16^ These studies reported that HP H^13^CO_3_^−^is sensitive to physiological perturbations in PDH flux,^9,17^ correlates with cardiomyopathies with manifest alteration in PDH activity,^9,10,18^ and reflects drug-induced changes in PDH flux.^17,19^

A ketogenic diet (KD) also alters cardiac glucose metabolism, inducing a metabolic shift towards the use of ketones for energy production.^20^ It has been reported that a KD has therapeutic effects on diseases such as epilepsy,^21,22^ PDH deficiency,^23,24^ and traumatic brain injury.^25^ Beyond patient managements, following a KD has become a trend to the general public, driven by its positive effects on weight loss for obese individuals^26^ and enhanced performance for endurance athletes.^27,28^ As a result, approximately 12.9 millions of Americans follow the KD each year.^29^ However, a KD increases low-density lipoprotein cholesterol in the blood^30,31^ and insulin resistance,^32^ having risks of developing metabolic syndrome and potential adverse effects on the function of the heart. Importantly, the long-term cardiovascular effects are unknown.^33^ Therefore, the reversibility of specific metabolic changes, induced by KD, needs to be investigated.

Metabolic effects and reprogramming associated with a KD have been studied,^34–36^ primarily based on *ex vivo* experiments. It was reported that a KD induces an increase in the content and activity of PDK4 and inhibition of PDH and glucose oxidation.^37^ However, reversibility and temporal dynamics of altered PDH activity induced by a KD is underexplored. In this study, we investigated the reversibility of myocardial PDH flux, suppressed by a KD, and its recovery dynamics when switching back to a normal diet (ND), using ^13^C MRS with HP [1-^13^C]pyruvate *in vivo*, and the *in vivo* results were compared with *ex vivo* enzymatic analysis.

## 2. Method

### 2.1 Overall study design and feeding condition

A total of 38 healthy male Sprague-Dawley rats (Charles River Laboratories, Houston, TX, USA) were used in this study and randomly assigned to subgroups. The animals were used for *in vivo* MRS, tissue isotopomer analysis, enzymatic analysis, or combination of these. The detailed usage of the animals is summarized in the Supplemental Information (Supplementary material online, *Table S1*). Inclusion of only male rats was to avoid biological variability that complicates the interpretation of metabolic adaptations to dietary interventions.^38^ Body weight was measured twice a week throughout the study. To minimize any confounders, all animals were ordered at the same age and housed in the same room. All studies were approved by the UT Southwestern Institutional Animal Care and Use Committee and conducted in accordance with the NIH Guide for the Care and Use of Laboratory Animals.

Two types of diet (Envigo Rms, Inc., Indianapolis, IN, USA) were used for the rats: the standard ND (Teklad Global Diet 2014, 13% kcal from fat, 67% kcal from carbohydrates, and 20% kcal from protein; 2.9 kcal/g) and the KD [Teklad Customer Diet, TD.96355, 90.5% kcal from fat (vegetable shortening and corn oil), 0.3% kcal from carbohydrates, and 9.1% kcal from protein; 6.7 kcal/g], which elevates levels of circulating β-hydroxybutyrate and ketones.^39,40^ Cardiac metabolism under three nutritional conditions was investigated: (i) ND, (ii) KD for 5 weeks, and (iii) reverted from a 5-week KD to ND. The KD was replenished three times a week. For each cage, the amount of diet consumed was calculated as 25 g × (number of rats per cage)×(days until the next refill). On each feeding day, both the total weight of the remaining food and the total weight of the newly added food were recorded to determine food intake. Although no animals met the criteria, those showing hypoactivity, dehydration, or severe weight loss (>20%) would have been excluded. Cardiac function was not monitored in this study, based on the weak correlation between KD-induced metabolic changes and function in the absence of cardiovascular disease.^41,42^

In addition, 20 healthy male rats were investigated for cross-sectional *ex vivo* enzymatic analyses of KD rats following a shorter KD duration (2 weeks) and subsequent diet reversal (2, 5, and 8 days) as well as age-matched controls.

### 2.2 *In vivo* MR experiments

Longitudinal imaging studies were performed to monitor KD-associated metabolic changes. Fifteen of the rats were used for *in vivo* experiments. The switch of diet started at 10 weeks of age (242–278 g). All control animals (CT group, *n* = 7) were fed with normal rodent chow, whereas the other group (KD group, *n* = 8) were fed with the KD (TD96355) meal for 5 weeks, immediately followed by the ND for 8 days. For imaging, each rat was continuously anaesthetized under fed condition using a vaporizer with 2–3% isoflurane in 1.5 L/min oxygen and the tail vein was cannulated with a catheter before positioning in the MRI scanner. Imaging data was collected between 8 am and 10 am to normalize the metabolic state. An integrated ^1^H/^13^C MRI protocol that includes a localizer, B_0_ shimming, and a slice-selective, time-resolved ^13^C MRS with a bolus injection of 120-mM HP [1-^13^C]pyruvate (1.5 mmol/kg body weight) via the tail vein catheter was used. Description on the MR protocol is included in the Supplemental Information. Missing datapoints (Rat IDs: 4, 27, 28) were due to equipment failure or maintenance.

### 2.3 Euthanasia

The rat was placed in home cage, which was used as the chamber for euthanasia. CO_2_ was delivered by turning on the CO_2_ tank and adjusting the flow rate to 3–11 L/min. The animal remained in the chamber for a minimum of 3–5 min, during which signs of cessation of vital functions, including limb movement, twitching, breathing, or pink skin colour, were observed. After removing the animal from the chamber, death was confirmed by the absence of breathing and a heartbeat. To ensure the animal’s death, a secondary method of euthanasia, thoracotomy, was performed.

### 2.4 Hyperpolarization of [1-^13^C]pyruvate

For dynamic nuclear polarization (DNP), a sample that contains 55.8 μL of neat 14-M [1-^13^C]pyruvic acid (Sigma-Aldrich, St. Louis, MO, USA) and 15-mM OX063 trityl radical (GE Healthcare, Waukesha, WI) was prepared in a sample vial. The sample vial was assembled to a research fluid path (GE Healthcare) and 16 mL of dissolution media (0.1 g/L EDTA-Na_2_) was loaded in the dissolution syringe of the fluid path. Each sample was polarized using a SPINlab™ DNP polarizer (GE Healthcare) for 3–4 h, rapidly dissolved with the preheated dissolution media, and neutralized with NaOH immediately before injecting to animals.^43^ The final solution was ∼6 mL of 120-mM [1-^13^C]pyruvate with pH of ∼7.5.

### 2.5 Nuclear magnetic resonance isotopomer analysis

For cross-sectional tissue nuclear magnetic resonance (NMR) analyses, three groups of Sprague-Dawley rats were used: CT group (ND for 5 weeks, *n* = 5), KD group (KD for 5 weeks, *n* = 4), and KD-to-ND reverted (RT) group (5-weeks KD followed by ND for 9 days, *n* = 4). To mimic the *in vivo* experiment, each rat was anaesthetized with 2–3% isoflurane and the tail vein was catheterized (same parameters as *in vivo* studies). The heart was collected between 9 and 10 am, 2 min after a bolus injection (injection rate = 0.25 mL/s) of a non-hyperpolarized mixture of 120-mM [1,2-^13^C_2_]acetate (1.5 mmol/kg) and 120-mM [3-^13^C]pyruvate (1.5 mmol/kg) through tail vein. The harvested heart tissue was immediately freeze-clamped and prepared for NMR isotopomer analysis as described in the Supplemental Information.

### 2.6 Measurement of mitochondrial PDH activity, phosphorylated PDH, and PDK4

Three groups of rats were used for tissue analysis: CT group (*n* = 3), KD group (*n* = 4), and RT group (*n* = 3). Rats were anaesthetized using a vaporizer with 3% isoflurane in 1.5 L/min and hearts were excised between 9 and 10 am and homogenized in ice-cold isolation buffer 10 mM 3-(N-Morpholino)propanesulfonic acid (MOPS), 1.0 mM EDTA, 210 mM mannitol, 70 mM sucrose, 20 mM NaF, and 5 mM dichloroacetate at pH 7.4 using a Polytron homogenizer. The homogenate was centrifuged at 500 × *g* for 5 min (4°C) and the supernatant was filtered through cheese cloth. The mitochondrial pellet was obtained by centrifugation of the supernatant at 12 000 × *g* for 10 min (4°C).^44^ Mitochondria were resuspended in homogenization buffer at a final concentration of 20–30 mg/mL. Protein determinations were made using the bicinchoninic acid method (Pierce™; Thermo Fisher Scientific, Waltham, MA, USA) with bovine serum albumin as standard. Mitochondria were then diluted to 0.05 mg/mL in buffer containing 25 mM MOPS and 0.05% Triton X-100 at pH 7.4. Solubilization of mitochondria with 0.05% Triton X-100 inhibits complex I of the respiratory chain preventing consumption of NADH. PDH activity was measured spectrophotometrically (Agilent, 8452A) as the rate of NAD^+^ reduction to NADH (340 nm, ε = 6200 M^−1^cm^−1^) upon addition of 2.5 mM pyruvate, 0.1 mM CoASH, 0.2 mM thiamin pyrophosphate, 1.0 mM NAD^+^, and 5.0 mM MgCl_2_ at pH 7.4.^44^

### 2.7 Western blot and CoASH analysis

PDH-E1α, phospho-PDH-E1α, and PDK4 antibodies were validated by Western blot detection of protein at the appropriate molecular weight and the requirement of primary antibody for secondary antibody binding.^45^ For CoASH analysis, approximately 30–50 mg of frozen tissue was extracted with perchloric acid, proteins were pelleted, and CoASH in the supernatants was analysed by ion-pair reverse-phase HPLC with UV/Vis detection at 254 nm. CoASH levels were quantified using a standard curve and expressed as nmol/mg protein. Detailed procedures are described in the Supplemental Information.

### 2.8 Post-processing and quantification of metabolites

**Figure 2:**
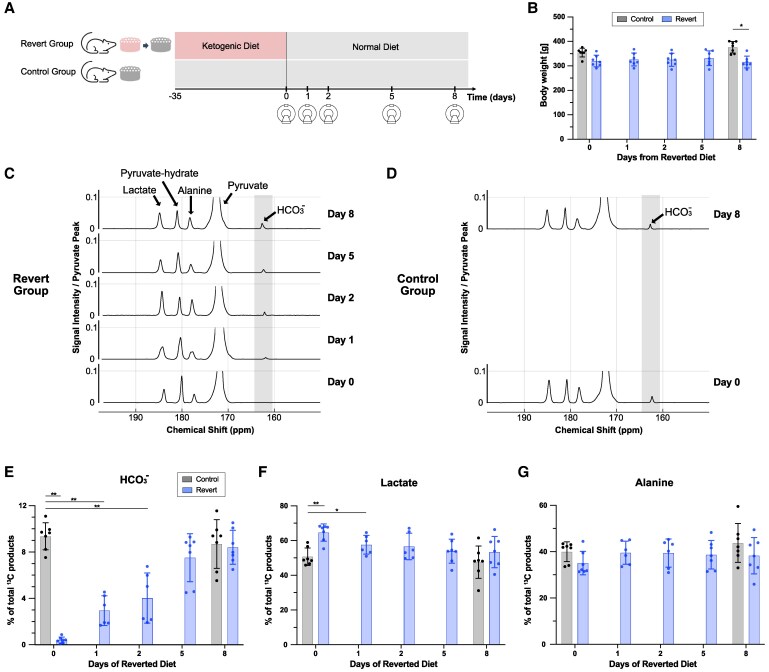
Recovery of *in vivo* myocardial PDH flux by reverting KD to ND. (*A*) *In vivo* imaging timeline for the KD-to-ND revert group (*n* = 7) and the ND-fed control group (*n* = 7). (*B*) Body weight monitored during the timeframe. (*C*) Time-averaged cardiac ^13^C spectra acquired from a representative rat at Day 0, 1, 2, 5, and 8 after changing the diet. Reverting to ND gradually recovered the PDH flux (H^13^CO_3_^−^/TP). (*D*) Age-matching CT group had non-significant changes. (*E*) HP H^13^CO_3_^−^/TP, (*F*) [1-^13^C]lactate/TP, and (*G*) [1-^13^C]alanine/TP were longitudinally monitored. Unpaired *t*-tests were used (two tailed, α = 0.05). * and ** indicate *P* < 0.05 and *P* < 0.01, respectively. TP, total HP ^13^C products.

*In vivo*  ^13^C raw data from the time-resolved slice-selective spectroscopy were processed using MATLAB (Mathworks, Natick, MA, USA; version 9.11.0.1809720). The data were first apodized by a 10-Hz Gaussian filter, followed by zero-filling by a factor of 4 and a fast Fourier transform. HP H^13^CO_3_^−^, [1-^13^C]lactate, [1-^13^C]alanine, [1-^13^C]pyruvate-hydrate, and [1-^13^C]pyruvate signals were quantified from the cumulative spectrum, summed over 0–90 s, by integrating the respective peak in absorption mode after a 0th order phase correction. The integrated metabolite peaks were normalized to the total HP ^13^C products (TP), which is the sum of HP H^13^CO_3_^−^, [1-^13^C]lactate, and [1-^13^C]alanine. The normalized HP metabolites were used to evaluate metabolic changes between groups or time points. Time-courses of H^13^CO_3_^−^, [1-^13^C]lactate, and [1-^13^C]alanine were measured in a similar manner from time-resolved spectra, then normalized to the TP (*Figure 1*). For display, each cumulative spectrum was normalized by the pyruvate peak (*Figures 1* and *2*). For a clear visual comparison of HP spectra between groups, the rolling baseline was subtracted by fitting a spline function to the signal-free regions of each spectrum (*Figure 2*).

NMR data were processed using TopSpin (Bruker, Billerica, MA, USA; version 4.3.0). After 0th and 1st order phase corrections, lactate and alanine peaks were measured from ^1^H spectra and glutamate C3 and C4 resonances were measured from ^13^C spectra. Fractional enrichments of lactate (1.31 ppm) and alanine (1.45 ppm) were calculated from J_CH_-coupled outer doublets relative to the central doublets. In ^13^C spectra, relative contribution of pyruvate towards acetyl-CoA was calculated by estimating the fractional concentration of [2-^13^C]acetyl-CoA ($F_{C2}$), which was generated from [3-^13^C]pyruvate, from C3 and C4 glutamate resonances.^46^ Similarly, relative contribution of acetate to acetyl-CoA [(1,2-^13^C_2_)acetyl-CoA] was calculated from the glutamate peaks by estimating the fractional concentration of [1,2-^13^C_2_]acetyl-CoA ($F_{C12}$). The following equations were used from glutamate peaks:


(1) $$F_{C2}=\int C4\;D34\cdot \frac{\int C4}{\int C3}$$

(2) $$F_{C12}=\int C4\;Q\cdot \frac{\int C4}{\int C3}$$

### 2.9 Plasma analysis

For plasma analysis, separate cohorts of a KD group (*n* = 5) and a control group (*n* = 5) were prepared. As in the HP study, the KD group was maintained on the KD for 5 weeks and then switched back to the standard diet. Blood samples were collected from both groups under fed condition at 10:30–11:30 am at baseline, and at 2, 3.5, and 5 weeks on the assigned diets. After reverting the KD, samples were obtained on the day of switching and at 2, 5, 8 days, as well as 2 weeks post-switch. Whole blood (1 mL) was collected from the tail vein of each rat into Eppendorf tubes, containing 20 µL of heparin (1000 U/mL) to prevent coagulation. Samples were immediately placed on ice and centrifuged at 2000 × *g* for 10 min at 4°C to separate plasma. The resulting plasma was analysed for circulating levels of ketone bodies, glucose, and free fatty acids. Total blood ketone levels were measured using the Wako Autokit Total Ketone Bodies assay (415–73301; Wako Chemicals, Lexington, MA). Non-esterified fatty acids (NEFAs) were quantified using the Wako NEFA kit (999–34691, 995–34791, 991–34891, 993–35191, 276–76491; Wako Chemicals). Glucose concentrations were measured using a VITROS 350 clinical analyzer (microchemical slides technology; Ortho Clinical Diagnostics, Raritan, NJ).

### 2.10 Data analysis and statistical evaluation

The results are reported as mean ± standard deviation. All data were analysed blinded to the group assignment of each animal. J.C., S.A.N., and Z.E. were aware of the group allocation until the interpretation and analysis of the results. For evaluating statistical significance, GraphPad Prism software (version 10.4.10) was used. Two-way ANOVA tests were performed to evaluate longitudinal metabolic differences between CT and KD groups at corresponding age. For comparing CT and RT and for evaluating *ex vivo* data of CT, KD, and RT groups, unpaired *t*-tests between the groups were used with two-tailed distributions and equal variances (α = 0.05), assuming normal distribution.^47^ A *P* value smaller than 0.05 was considered statistically significant and marked as a single asterisk (*). *P* values smaller than 0.01 are indicated with double asterisks (**).

## 3. Results

KD-fed rats consumed 13.0 ± 2.9 g (87.1 ± 19.4 kcal) of food daily, which is comparable to the intake of rats with a normal diet,^48^ except the first day of KD (6.6 g), Supplementary material online, *Figure S1*. The KD group experienced short-term weight loss (*P* = 1.1 × 10^−3^, *Figure 1, B*), which was consistent with other KD studies.^49–51^ After a 2-week KD, HP H^13^CO_3_^−^ produced in the heart decreased from 8.56 ± 2.29% of the total HP products (TP) at baseline (*P* = 0.97 vs. ND group) to 0.37 ± 0.34% (*P* = 1.9 × 10^−9^ vs. ND group), with little change observed with extended diet durations (5 weeks; 0.46 ± 0.27%, *P* = 1.5 × 10^−9^*vs.* ND group), *Figure 1, C–E*. Conversely, *in vivo* HP [1-^13^C]lactate/TP was higher in the KD at 2 weeks (*P* = 6.2 × 10^−3^) and 5 weeks (*P* = 1.9 × 10^−3^) compared to the ND group, *Figure 1, F*. Within the KD group, lactate/TP remained relatively stable (58.21 ± 3.95% at baseline, 59.56 ± 2.73% at 2 weeks, 65.46 ± 5.28% at 5 weeks), whereas in the ND group it decreased with age (61.61 ± 3.48% at baseline, 46.67 ± 7.84% at 2 weeks, 51.04 ± 5.42% at 5 weeks). This pattern is consistent with an age-related decline in myocardial LDH activity.^52^ It is worth noting that the overall large proportion of lactate is partly due to the significant contribution of extracellular HP lactate signals originating from plasma or other organs such as the liver^53,54^ in addition to accelerated isotope exchange between HP pyruvate and unlabelled lactate pool.^55^ HP [1-^13^C]alanine/TP did not show significant differences between CT and KD groups (*P* = 0.86 at baseline, *P* = 0.76 at 2 weeks, and *P* = 0.48 at 5 weeks), *Figure 1, G*. Longitudinal changes of *in vivo* HP metabolites during KD and ND are summarized in Supplemental Information (Supplementary material online, *Table S2*).

Reverting to ND did not show noticeable change in body weight, maintaining the weight difference induced by the KD (*Figure 2, B*). However, diet reversal gradually recovered the PDH flux (H^13^CO_3_^−^/TP) from 0.40 ± 0.26% (Day 0) to 2.94 ± 1.29% (Day 1), 4.02 ± 2.16% (Day 2), 7.51 ± 2.07% (Day 5), and 8.40 ± 1.47% (Day 8), *Figure 2, C-E*. Age-matching CT group had non-significant changes from 9.36 ± 1.17% to 8.69 ± 2.10% (*P* = 0.36) during the same period. In contrast, [1-^13^C]lactate/TP in the KD group showed the opposite but similar dynamic patterns, likely reflecting the redirected metabolic fate of pyruvate towards PDH (*Figure 2, F*). Again, [1-^13^C]alanine/TP did not show noticeable changes and was relatively unchanged after reverting from KD to ND (*Figure 2, G*). Longitudinal changes of *in vivo* HP product levels after reverting to ND are summarized in Supplemental Information (Supplementary material online, *Table S3*).

**Figure 3:**
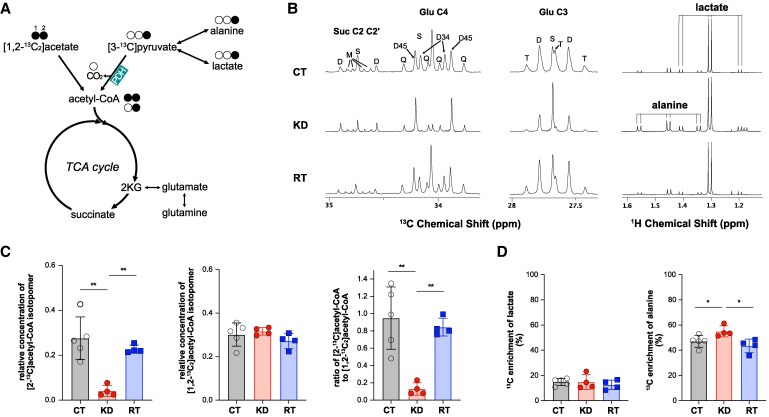
NMR isotopomer analysis using [3-^13^C]pyruvate and [1,2-^13^C_2_]acetate. (*A*) Metabolic pathways of labelled substrates. (*B*) Representative ^13^C (27.0–35.0 ppm) and ^1^H (1.1–1.6 ppm) NMR spectra from control (CT, *n* = 5), ketogenic diet (KD, *n* = 4), and reverted (RT, *n* = 4) rat hearts. (*C*) Relative concentrations of [2-^13^C]acetyl-CoA isotopomer and [1,2-^13^C_2_]acetyl-CoA were calculated from ^13^C NMR. (*D*) ^13^C enrichments of lactate and alanine were calculated from ^1^H NMR. Unpaired *t*-tests were used (two tailed, α = 0.05). * and ** indicate *P* < 0.05 and *P* < 0.01, respectively. Suc, succinate; Glu, glutamate; CT, control; KD, ketogenic diet; RT, reverted diet.

Separately, a snapshot of *in vivo* metabolism was assessed via isotopomer NMR analysis from freeze-clamped cardiac tissues of KD, RT, and CT groups to confirm the *in vivo* measurements. Bolus-injected [3-^13^C]pyruvate and [1,2-^13^C_2_]acetate generated [2-^13^C]acetyl-CoA and [1,2-^13^C_2_]acetyl-CoA, respectively (*Figure 3, A*). Isotopomer analysis of glutamate C4 resonance showed that relative concentration of [2-^13^C]acetyl-CoA isotopomer to the total acetyl-CoA was reduced in the KD group and recovered in the RT group. Relative contribution of the injected pyruvate to acetyl-CoA was 4.1 ± 2.5% for KD, 22.7 ± 1.82% for RT, and 27.6 ± 9.5% for CT (*Figure 3, B* and *C*). Relative contribution of the injected acetate to acetyl-CoA was comparable between groups (31.6 ± 1.7% for KD, 37.2 ± 6.4% for RT, 30.2 ± 5.4% for CT). Fractional ^13^C enrichment in lactate, measured from ^1^H NMR, was also comparable between groups (14.8 ± 5.9 for KD, 12.6 ± 3.8% for RT, 14.8 ± 2.8% for CT). Two pairs of outer doublets observed in the lactate resonance region represent the three-bond J_CH_ spin–spin coupling between methyl protons and the methyl ^13^C, confirming the presence of injected lactate in the metabolite pool. Fractional ^13^C enrichment in alanine was higher in the KD group (55.0 ± 4.4%) as compared with RT (43.5 ± 5.4%, *P* = 0.016) and CT (47.1 ± 4.8%, *P* = 0.038) as summarized in *Figure 3, B* and *D*, likely due to elevated circulating alanine aminotransferase.^56,57^ The NMR measurements are summarized in the Supplemental Information (Supplementary material online, *Table S4*).

**Figure 4:**
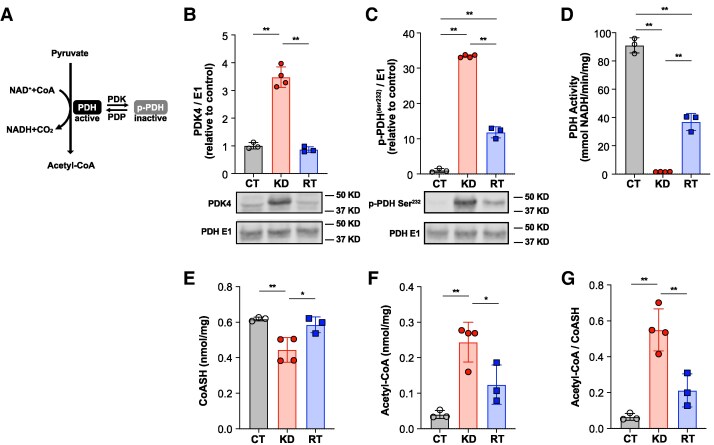
Enzymatic analysis of PDH. (*A*) Active and inactive forms of PDH are regulated by PDK and PDP. (*B*) PDK, (*C*) PDH activity, (*D*) phosphorylated PDH (p-PDH), (*E*) CoASH, (*F*) Acetyl-CoA, and (*G*) Acetyl-CoA/CoASH levels were measured *ex vivo* from the cardiac tissue of the control (CT, *n* = 3), ketogenic diet (KD, 5 weeks, *n* = 4), and KD-to-ND reverted (RT, 5 weeks of KD followed by 9 days of ND, *n* = 3) groups. Unpaired *t*-tests were used (two tailed, α = 0.05). * and ** indicate *P* < 0.05 and *P* < 0.01, respectively. PDH, pyruvate dehydrogenase; PDK, pyruvate dehydrogenase kinase; PDP, pyruvate dehydrogenase phosphatase.

Enzymatic analysis was performed from cardiac tissues of KD, RT, and CT groups to compare with the *in vivo* observation. The *ex vivo* analysis revealed that PDK4 level in the myocardium was elevated with KD (3.48 ± 0.37, relative to control), then returned to the normal level (0.87 ± 0.10) at Day 9 after reverting to ND (*Figure 4, B*). On the contrary, PDH activity and phosphorylated PDH level were not fully recovered to the normal level (*Figure 4, C* and *D*). PDH activity in the cardiac tissue increased from 1.60 ± 0.12 mmol NADH/min/mg in KD group (*n* = 4) to 36.80 ± 6.07 in RT group (*n* = 3), which was significantly lower than PDH activity in CT group (*n* = 3, 90.97 ± 5.40, *P* = 7.1 × 10^−5^). Similarly, phosphorylated PDH (*P*-PDH), the inactive form of PDH, was 33.39 ± 0.42-fold higher in KD as compared to CT but remained much higher level in RT (11.80 ± 1.58). The CoASH level was lower in the KD group (0.444 ± 0.069 nmol/mg) as compared to the CT group (0.618 ± 0.011, *P* = 0.008) and the RT group (0.585 ± 0.044, *P* = 0.028). The acetyl-CoA level and the acetyl-CoA-to-CoASH ratio showed the opposite pattern (*Figure 4, E-G*). The PDH measurements and the metabolite levels are summarized in the Supplemental Information (Supplementary material online, *Table S5*). These results contrasted with the complete recovery of both PDK4 and phosphorylated PDK at Day 8 after reverting to ND following a 2-week KD, Supplementary material online, *Figure S2*, suggesting a strong dependence of PDH recovery dynamics on the duration of KD exposure.

**Figure 5:**
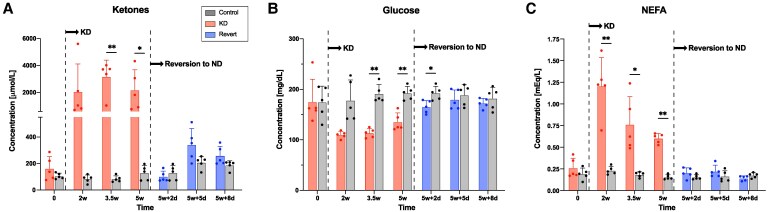
Plasma levels of ketones, glucose, and NEFA. Circulating ketones, glucose, and NEFA were measured from blood longitudinally at baseline, during KD up to 5 weeks, and after reversion to ND up to 8 days. (*A*) The elevated level of ketones by a KD remained consistent during the KD and decreased to the control level within 2 days of reverted diet. (*B*) Glucose level dropped with a KD, then recovered to the control level within 2 days of reverted diet. (*C*) The NEFA level peaked at 2 weeks of KD then gradually decreased over the period of KD, then decreased to the control level within 2 days of reverted diet. Two-way ANOVA tests were used (α = 0.05). * and ** indicate *P* < 0.05 and *P* < 0.01, respectively. KD, ketogenic diet; ND, normal diet; NEFA, non-esterified fatty acids.

Circulating profiles of ketones, glucose, and NEFAs in blood were shifted with the KD, *Figure 5*. The plasma ketone level increased by 12.10 ± 8.09-fold after two weeks of KD (baseline: 163.0 μmol/L ± 89.4, 2 weeks: 2057.8 ± 2061.2 μmol/L) while the control group showed no significant change (*P* = 0.13). The elevated ketone level in the KD group remained significant at 3.5 (3177.5 ± 1217.1, *P* = 4.7 × 10^−3^) and 5 weeks of KD (2180.0 ± 1508.9, *P* = 0.038) compared with the control group (84.6 ± 29.1 at 3.5 weeks, 84.3 ± 23.5 at 5 weeks). In contrast, glucose level showed the opposite trend, decreasing by 34.3 ± 14.9% at 2 weeks (110.0 ± 7.0 mg/dL) relative to baseline (174.5 ± 32.0 mg/dL). The reduced glucose level remained lower in the KD group at 3.5 weeks (113.5 ± 9.1, *P* = 1.4 × 10^−4^) and 5 weeks (135.5 ± 17.7, *P* = 5.7 × 10^−3^) than in the controls (191.5 ± 18.1 at 3.5 weeks, 192.5 ± 13.5 at 5 weeks). The KD also elevated plasma NEFAs by 5.53 ± 2.95-fold from baseline (0.262 ± 0.113 mEq/L) to 2 weeks (1.206 ± 0.330), which then gradually decreased over time to 0.764 ± 0.320 at 3.5 weeks and 0.603 ± 0.057 at 5 weeks. Nevertheless, NEFA levels remained significantly higher in the KD group than in controls (*P* = 2.5 × 10^−3^ at 2 weeks, 1.5 × 10^−2^ at 3.5 weeks, 6.0 × 10^−6^ at 5 weeks). Upon reversion to the ND, both ketone and NEFA levels returned to control levels within 2 days. However, glucose level recovered more slowly, remaining lower than controls at 2 days (*P =* 0.011) but fully recovering thereafter (*P* > 0.4).

## 4. Discussion

In this study, we show that myocardial PDH flux, measured by HP [1-^13^C]pyruvate *in vivo*, requires approximately 1 week to fully recover from a 5-week KD. Cross-sectional isotopomer analysis of freeze-clamped tissue using ^13^C NMR with [3-^13^C]pyruvate and [1,2-^13^C_2_]acetate supports this full recovery of myocardial PDH flux 1 week after reverting the diet. In contrast, *ex vivo* enzymatic analysis shows incomplete recovery of PDH activity during the same period.

Prolonged exposure to a KD induces a state of metabolic inflexibility in the heart, where cardiac ketolysis and β-oxidation predominate over carbohydrate oxidation. This metabolic shift reflects functional adaptions of associated transporters and enzymes for sustained utilization of ketones and fatty acids, consistent with the elevated plasma ketones and fatty acid levels and the reduced glucose concentrations observed here. Similar changes have been reported in KD-fed mice for 3 weeks, showing increased palmitate oxidation, unaltered β-hydroxybutyrate oxidation, and impaired glucose oxidation.^41^ Gallop *et al.* also reported that long-term KD also elevated circulating lipids and suppressed insulin secretion.^58^

The discrepancies between the ^13^C results and enzymatic analyses regarding the extent of recovery 1 week after reverting from a KD to an ND likely arise from the methodological differences. Due to complex metabolic cross talks and signalling pathways, metabolic fluxes need to be assessed under strict, well-controlled environments. *Ex vivo* enzyme assay measures the maximum enzyme activity. However, in physiological settings, metabolic systems typically operate below their maximal capacity, allowing for metabolic flexibility to maintain homeostasis. For instance, the activity of PDH depends on multiple factors, including malonyl-CoA levels, CoASH availability, PDK regulation, redox state, and alternative substrate pathways such as fatty acid oxidation.^59^ Enzymatic analyses of isolated cardiac mitochondria post-KD reversion revealed a reduction in PDK4 levels to those seen in control diet-fed rats. Despite this, PDH activity remained attenuated. Similarly, while acetyl-CoA levels were significantly lower, free CoASH levels returned to normal, suggesting that while acetyl-CoA synthesis or utilization was altered, the pool of available CoASH was restored.

In contrast, isotopomer analysis offers a more direct method of measuring *in vivo-*like metabolic fluxes. Using freeze-clamped tissues and substrates labelled with stable isotopes, NMR or mass spectrometry can capture metabolism under conditions closer to physiological states. In our study, we employed ^13^C-pyruvate and NMR to assess PDH flux, and the results showed fully recovered PDH flux, consistent with *in vivo* findings using HP [1-^13^C]pyruvate. Together, these findings suggest that, about 1 week after reverting the KD, myocardial PDH flux reaches the restored maximum PDH activity. However, the persistence of altered metabolic flexibility indicates incomplete recovery of the broader metabolic network. The discordance between enzymatic analyses and *in vivo*  ^13^C NMR results highlights the necessity of complementary approaches. Therefore, measuring metabolic fluxes *in vivo* under physiological conditions provides critical insights into dynamic metabolic states, which are often missed by *ex vivo* analyses. This integrative approach is essential for capturing the complexity of metabolic recovery.

Sustained enhancement of fatty acid oxidation may partly explain the delayed PDH recovery observed here. We previously showed that high-fat diet-induced declines in cardiac PDH activity plateau within 1 day, coinciding with increased PDK4 expression and PDH E1α phosphorylation.^44^ PDK4 is selectively degraded by the LON protease in a CoA-dependent manner upon dissociation from the PDH complex.^45^ However, even after normalization of PDK4, PDH can remain sensitized to fatty acid-induced inhibition due to persistent up-regulation of β-oxidation enzymes, including very long chain acyl-CoA dehydrogenase, carnitine palmitoyltransferase 2, and β-hydroxyacyl-CoA dehydrogenase.^60^ Moreover, elevated acetyl-CoA and ketone levels during KD can increase protein acetylation, which in turn may influence transcriptional regulation of metabolic enzymes. Such epigenetic or acetylation-mediated remodelling may also prolong metabolic reprogramming beyond the time course of PDH phosphorylation changes.

The slow recovery of PDH flux and metabolic flexibility resonates the challenges of accurately characterizing diseased-induced metabolic profiles, particularly when dietary interventions introduce significant metabolic shifts. Non-invasive assessment of disease-induced metabolic changes is crucial for diagnosing and managing cardiovascular diseases. Understanding how dietary conditions affect metabolism is important for developing clinically applicable imaging protocols. Clinically applicable *in vivo* imaging methodologies that measure carbohydrate metabolism, such as [^19^F]FDG-PET,^61^ deuterium metabolic imaging (DMI),^62^ and HP [^13^C]pyruvate MRI^63^ are highly sensitive to special diets that suppress glycolysis and pyruvate oxidation, such as KD.^64^ Consequently, the metabolic shifts induced by a KD often present challenges for assessing disease-specific cardiac metabolism. A common strategy is to administer a standardized diet to the subject prior to imaging. For instance, clinical protocols using FDG attempt to standardize glucose uptake by oral glucose loading protocol, which consists of 6–12 h fasting, glucose loading, and insulin administration prior to PET imaging.^65^ This is to maximize physiological PDH flux from a standardized (fasted) minimum baseline. Similarly, for human cardiac imaging studies with HP ^13^C-pyruvate, an oral glucose load after an overnight fast has been employed as a standardized preparation procedure^13–15^ due to its effectiveness in maximizing HP H^13^CO_3_^−^ production.^54^ However, the metabolic recovery rate of suppressed PDH activity has been unknown.^66^ Although diet-induced increases in PDK4 content are rapidly reversible,^44^ returning to a carbohydrate-based diet after a period of consuming a KD has post-diet effects on caloric intake, body weight gain, and insulin levels.^67^ Our study results confirms that the PDH flux, suppressed by a long-term KD, exhibited a protracted recovery in the myocardium after the diet was reverted, and therefore, an oral glucose challenge may not be sufficient to normalize the dietary component.

The prolonged PDH inactivation highlights the potential for lingering metabolic inflexibility following ketogenic interventions, which may have clinical relevance in conditions such as heart failure or diabetes, where PDH regulation is already impaired. However, recovery kinetics may differ in humans, given their slower basal metabolic rate^68^ and greater substrate flexibility, and higher reliance on carbohydrate oxidation,^69,70^ compared to rodents. Moreover, the abundance and turnover rate of mitochondrial enzymes, including PDH, are lower in humans,^71^ suggesting a slower onset but potentially faster recovery metabolic reprogramming once established.

Limitations were identified in this study, which suggest opportunities for future investigations. First, HP [1-^13^C]pyruvate was limited to assess PDH flux only up to acetyl-CoA formation since the labelled carbon is decarboxylated by PDH. Downstream metabolic fate of acetyl-CoA and contributions from other substrates were not explored. To link the PDH flux to pyruvate oxidation and to assess how acetyl-CoA is metabolized in the heart *in vivo*, HP [2-^13^C]pyruvate can be considered.^19,72^ Alternatively, HP [1-^13^C]acetyl-L-carnitine can be utilized for probing acetyl-CoA metabolism by detecting [5-^13^C]glutamate.^73^ Integrating HP [1-^13^C]pyruvate with additional HP substrates such as [1-^13^C]butyrate and [3-^13^C]acetoacetate may further delineate substrate competition.^74^ In addition, a fluxomic analysis using ^13^C-glucose could elucidate how glucose uptake and glycolysis recover relative to PDH flux since glucose fate in the myocardium can be influenced by elevated ketone availability.^75,76^ Second, each HP product was quantified as a relative amount compared to total HP ^13^C products. This metric is not sensitive to changes in the total product amounts. Although not done in our study due to the absence of polarization measurements, a potential solution for more consistent quantification between the modalities is normalization of each HP signals to its polarization level at the time of injection.^77^ Third, inclusion of only males limits translational relevance as sex-specific differences in PDH regulation are significant.^38,78^ Finally, while the current study focused on PDK4-mediated PDH regulation, other mechanisms that could diminish PDH activity in response to high dietary fat were not investigated. For instance, Ca^2+^ is known to activate PDH by stimulating pyruvate dehydrogenase phosphatase 1 and dephosphorylation of the enzyme. Future studies combining transcriptomic and acetyl-proteomic profiling will clarify these longer-term mechanisms.

In conclusion, KD-induced suppression of myocardial PDH flux is reversible but recovers slowly upon reintroduction of a ND. *In vivo* measurements of restored PDH flux aligned with PDK4 normalization but were not consistent with tissue level PDH activity, implying persistent metabolic inflexibility in the myocardium. These findings highlight the value of *in vivo* assessments of PDH activity for assessing cardiac carbohydrate metabolism and emphasize the complementary insights gained from integrating *in vivo* HP ^13^C MR and *ex vivo* biochemical analyses.

Translational perspective Such PDH inactivation may have clinical implications for heart failure or diabetes, where PDH regulation is impaired. Translating these results to humans requires consideration of slower metabolic rates, greater substrate flexibility, and higher reliance on carbohydrate oxidation, compared to rodents.

## Supplementary Material

cvag053_Supplementary_Data

## Data Availability

The data underlying this article will be shared on reasonable request to the corresponding author.
