# Supplementary material for: In vivo assessment of the recovery of myocardial pyruvate dehydrogenase activity following a ketogenic diet
Source: Cardiovasc Res. 2026 Feb 26;122(6):723–33. doi: 10.1093/cvr/cvag053 (PMC13019313; doi:10.1093/cvr/cvag053)
Supplement: cvag053_Supplementary_Data [file cvag053_supplementary_data.pdf]

**Supplemental Information**

**Title**: *In vivo* assessment of the recovery of myocardial pyruvate dehydrogenase activity following a ketogenic diet

**Authors and Affiliations**:
Jun Chen, Ph.D.[1], Zohreh Erfani, M.D.[1], Abdallah Elnwasany, M.D.[2,3], Sarah Al Nemri, B.A.[1], Joseph Park, B.A.[1], Mai T. Huynh, Ph.D.[1], Maheen Zaidi, B.S.[1], Crystal E. Harrison, Ph.D.[1], Xiaodong Wen, M.D.[1], Luke I. Szweda, Ph.D.[2,4], Jae Mo Park, Ph.D.[1,5,6,7]*

[1]Advanced Imaging Research Center, UT Southwestern Medical Center, Dallas TX 75390
[2]Division of Cardiology, Department of Internal Medicine, UT Southwestern Medical Center, Dallas TX 75390
[3]Department of Biology, University of Dallas, Irvin TX 75062
[4]Department of Medicine, University of Arizona, Tucson AZ 85719
[5]Department of Biomedical Engineering, UT Southwestern Medical Center, Dallas TX 75390
[6]Department of Radiology, UT Southwestern Medical Center, Dallas TX 75390
[7]Charles and Jane Pak Center for Mineral Metabolism and Clinical Research, UT Southwestern Medical Center, Dallas TX 75390

**Short Title**: Recovery of PDH activity after a ketogenic diet

* **Correspondence to:** Jae Mo Park, Ph.D.
The University of Texas Southwestern Medical Center
5323 Harry Hines Blvd. Dallas TX 75390
+1-214-645-7206, jaemo.park@utsouthwestern.edu

## Supplemental Figures

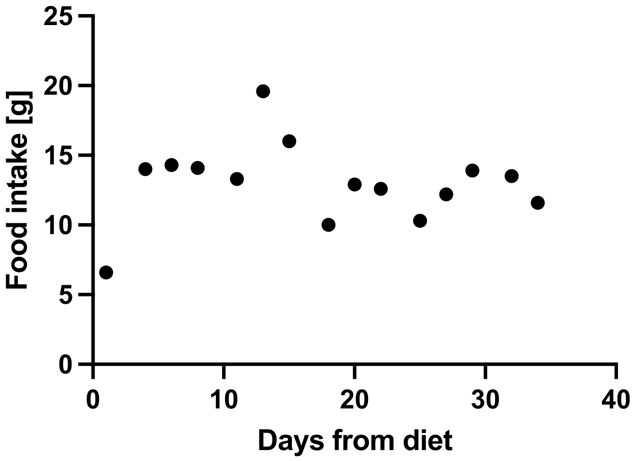


**Figure S1. Daily intake of KD.** Rats were fed a ketogenic diet (Teklad Custom Diet TD.96355; 90.5% kcal from fat [vegetable shortening and corn oil], 0.3% kcal from carbohydrates, and 9.1% kcal from protein; 6.7 kcal/g). The average daily intake per rat was consistent throughout the 5-week feeding period, except for the first day (6.6 g), after which consumption stabilized.

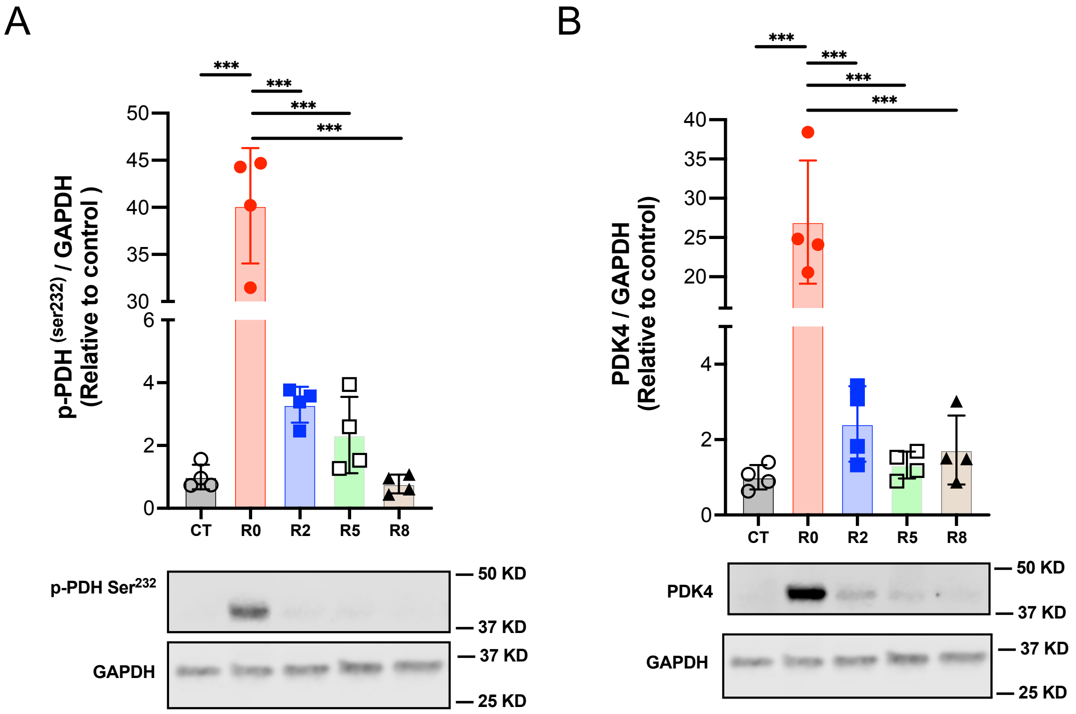


**Figure S2. Recovery PDK4 and p-PDH after 2 weeks of KD.** (A) PDK4 and (B) phosphorylated PDH (p-PDH) were measured *ex vivo* from the cardiac tissue of the control group (CT, n = 4), the ketogenic diet group (KD, 2 weeks, n = 4), and the KD-to-ND reverted (RT, 2 weeks of KD followed by ND) groups, measured at 2-day (n = 4), 5-day (n = 4), and 8-day (n = 8) from the reversion. Tukey's multiple comparison tests were used ($\alpha = 0.05$). *** indicates $P < 0.001$. PDH, pyruvate dehydrogenase; PDK, pyruvate dehydrogenase kinase.

(A)

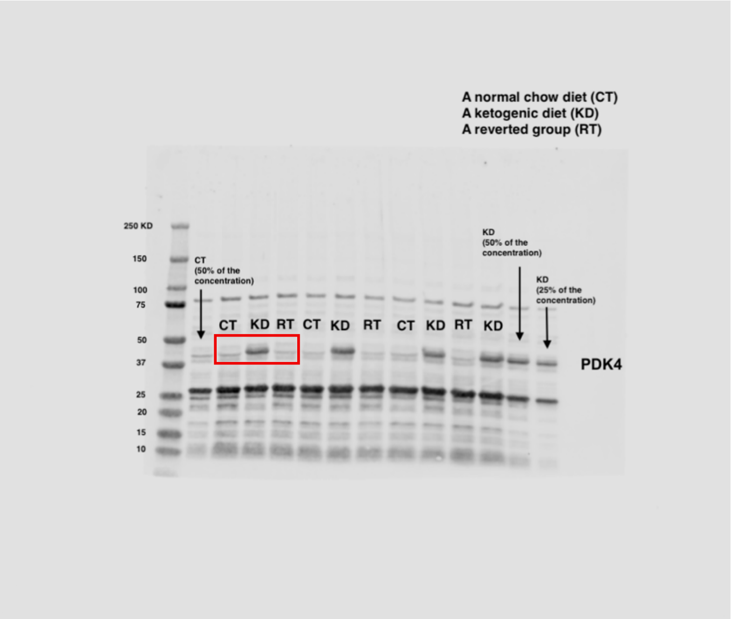


(B)

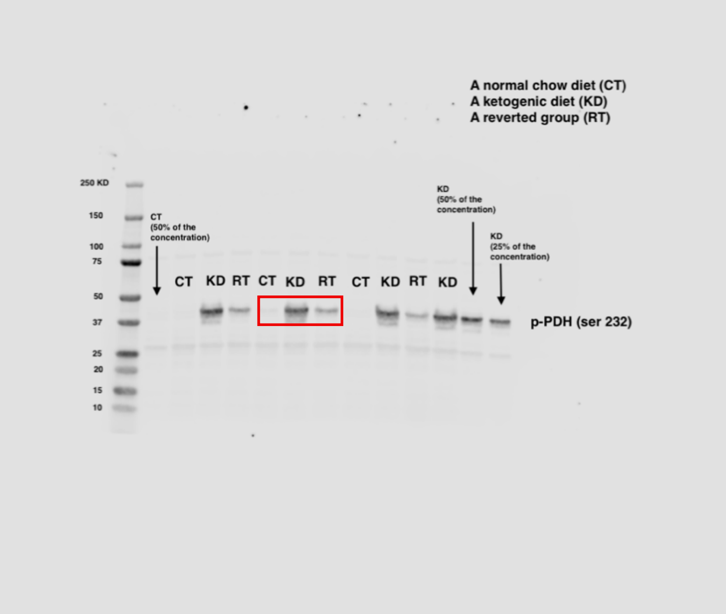


(C)

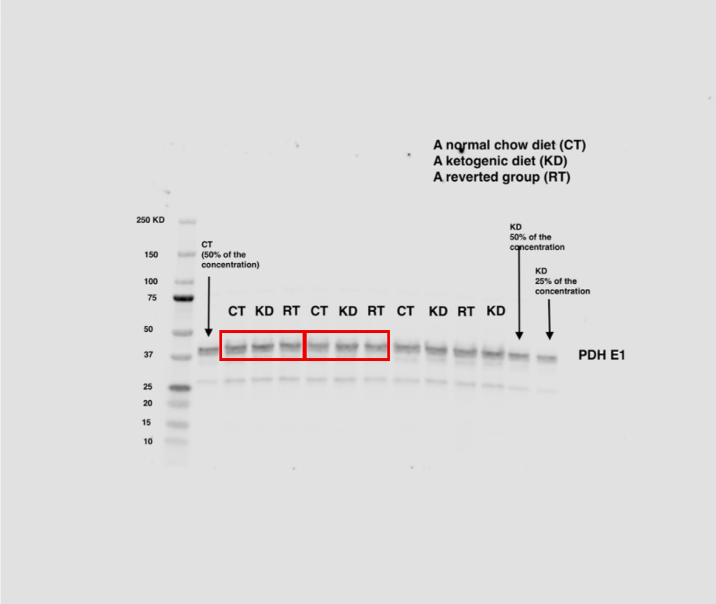


**Figure S3. Complete gels of Figure 4.** (A) PDK4, (B) p-PDH Ser$^{232}$, (C) PDH E1. Red boxes indicate the cropped blot images.

(A)
(B)
(C)

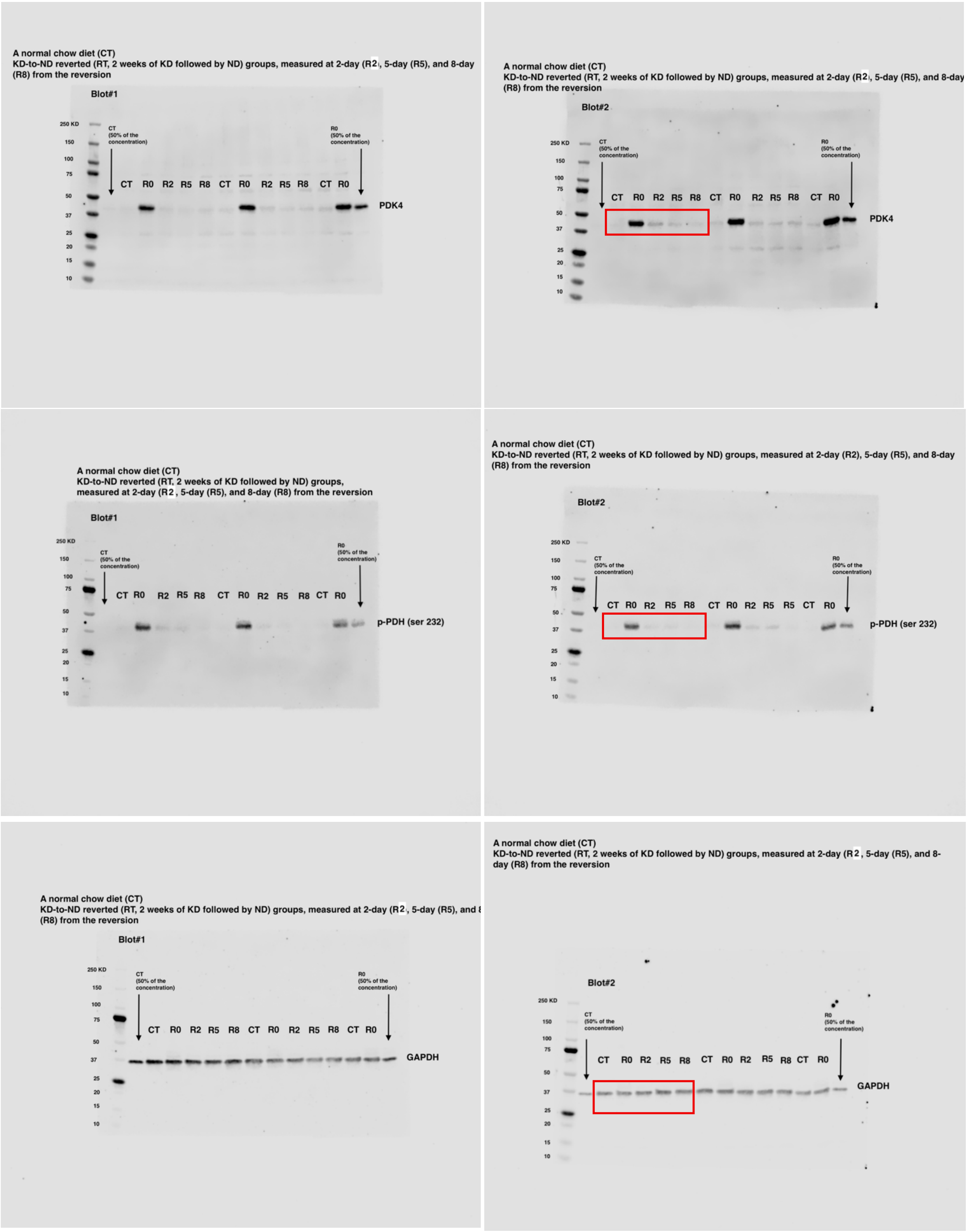


**Figure S4. Complete gels of Figure S2.** (A) PDK4, (B) p-PDH Ser$^{232}$, (C) GAPDH. Red boxes indicate the cropped blot images.

## Supplemental Tables

**Table S1. Animal usage.** KD: ketogenic diet; ND: normal diet; RT: reverted diet (5 weeks of KD followed by 8 days of ND).

| ID | *In vivo* imaging | | | | | | | | | | *Ex vivo* | |
|---|---|---|---|---|---|---|---|---|---|---|---|---|
| | Baseline | ND | | | KD | | RT | | | | NMR | PDH analysis |
| | | 2w | 5w | 5w + 8d | 2w | 5w | 1d | 2d | 5d | 8d | | |
| 01 | × | - | - | - | × | × | × | × | × | × | RT | - |
| 02 | × | - | - | - | × | × | × | × | × | × | RT | - |
| 03 | × | - | - | - | × | × | × | × | × | × | RT | - |
| 04 | × | - | - | - | × | × | × | - | × | × | RT | - |
| 05 | - | - | - | - | - | - | - | - | - | - | KD | - |
| 06 | - | - | - | - | - | - | - | - | - | - | KD | - |
| 07 | - | - | - | - | - | - | - | - | - | - | KD | - |
| 08 | - | - | - | - | - | - | - | - | - | - | KD | - |
| 09 | - | - | - | - | - | - | - | - | - | - | ND | - |
| 10 | - | - | - | - | - | - | - | - | - | - | ND | - |
| 11 | - | - | - | - | - | - | - | - | - | - | ND | - |
| 12 | - | - | - | - | - | - | - | - | - | - | - | ND |
| 13 | × | × | × | × | - | - | - | - | - | - | ND | - |
| 14 | × | × | × | × | - | - | - | - | - | - | - | - |
| 15 | × | × | × | × | - | - | - | - | - | - | - | - |
| 16 | × | × | × | × | - | - | - | - | - | - | - | - |
| 17 | - | - | - | - | - | - | - | - | - | - | ND | - |
| 18 | - | - | - | - | - | - | - | - | - | - | - | KD |
| 19 | - | - | - | - | × | × | - | - | - | - | - | KD |
| 20 | - | - | - | - | - | - | - | - | - | - | - | KD |
| 21 | - | - | - | - | - | - | - | - | - | - | - | KD |
| 22 | - | - | - | - | - | × | × | × | × | × | - | RT |
| 23 | - | - | - | - | - | × | × | × | × | × | - | RT |
| 24 | - | - | - | - | - | - | - | - | - | - | - | RT |
| 25 | - | - | - | - | - | × | - | × | × | × | - | - |
| 26 | × | × | × | × | - | - | - | - | - | - | - | ND |
| 27 | - | × | × | × | - | - | - | - | - | - | - | ND |
| 28 | - | - | × | × | - | - | - | - | - | - | - | - |
| | | | | | | | | | | | | |

| ID | Blood analysis | | | | | | | | | | | | |
|---|---|---|---|---|---|---|---|---|---|---|---|---|---|
| | Baseline | ND | | | | | | KD | | | RT | | |
| | | 2w | 3.5 w | 5w | 5w+ 2d | 5w+ 5d | 5w+ 8d | 2w | 3.5 w | 5w | 5w+ 2d | 5w+ 5d | 5w+ 8d |
| 29-33 | × | × | × | × | × | × | × | - | - | - | - | - | - |
| 34-38 | × | - | - | - | - | - | - | × | × | × | × | × | × |

**Table S2.** Longitudinal HP $^{13}C$ metabolite levels during KD and ND.

| Diet | Metabolite | Baseline | 2 weeks | 5 weeks |
|---|---|---|---|---|
| KD | $HCO_3^-$/TP (%) | 8.56 ± 2.29 | 0.37 ± 0.34 | 0.46 ± 0.27 |
| | Lactate/TP (%) | 58.21 ± 3.95 | 59.56 ± 2.73 | 65.46 ± 5.28 |
| | Alanine/TP (%) | 33.23 ± 2.73 | 40.07 ± 1.78 | 34.08 ± 0.38 |
| ND | $HCO_3^-$/TP (%) | 9.28 ± 2.31 | 9.10 ± 0.81 | 9.30 ± 1.27 |
| | Lactate/TP (%) | 61.61 ± 3.48 | 46.67 ± 7.84 | 51.04 ± 5.42 |
| | Alanine/TP (%) | 29.11 ± 3.89 | 44.23 ± 7.85 | 39.67 ± 4.58 |

**Table S3.** Longitudinal HP $^{13}C$ metabolite levels measured when reverted from KD to ND.

| Diet | Metabolite | Day 0 | Day 1 | Day 2 | Day 5 | Day 8 |
|---|---|---|---|---|---|---|
| RT | $HCO_3^-$/TP (%) | 0.40 ± 0.26 | 2.94 ± 1.29 | 4.02 ± 2.16 | 7.51 ± 2.07 | 8.40 ± 1.47 |
| | Lactate/TP (%) | 64.58 ± 4.94 | 57.56 ± 5.41 | 56.61 ± 7.61 | 53.83 ± 6.89 | 53.36 ± 9.01 |
| | Alanine/TP (%) | 35.02 ± 1.28 | 39.50 ± 4.75 | 39.37 ± 7.24 | 38.66 ± 4.36 | 38.24 ± 6.59 |
| CT | $HCO_3^-$/TP (%) | 9.36 ± 1.17 | - | - | - | 8.69 ± 2.10 |
| | Lactate/TP (%) | 50.67 ± 5.04 | - | - | - | 47.54 ± 9.29 |
| | Alanine/TP (%) | 39.98 ± 4.26 | - | - | - | 43.76 ± 8.42 |

**Table S4. *Ex vivo* NMR measurements.** KD: ketogenic diet; ND: normal diet; RT: reverted diet (5 weeks of KD followed by 9 days of ND).

| | CT | KD | RT |
|---|---|---|---|
| [2-$^{13}$C]acetyl-CoA/total acetyl-CoA | 0.2757 ± 0.0947 | 0.0412 ± 0.0246 | 0.2273 ± 0.0182 |
| [1,2-$^{13}C_2$]acetyl-CoA/total acetyl-CoA | 0.3016 ± 0.0537 | 0.3161 ± 0.0170 | 0.2718 ± 0.0352 |
| [2-$^{13}$C]acetyl-CoA/[1,2-$^{13}C_2$]acetyl-CoA | 0.9478 ± 0.3600 | 0.1291 ± 0.0717 | 0.8444 ± 0.1026 |
| $^{13}$C-enrichment in lactate | 0.1482 ± 0.0283 | 0.1482 ± 0.0588 | 0.1257 ± 0.0376 |
| $^{13}$C-enrichment in alanine | 0.4710 ± 0.0475 | 0.5497 ± 0.0436 | 0.4345 ± 0.0544 |

**Table S5. *Ex vivo* PDH analysis and metabolite levels.** KD: ketogenic diet; ND: normal diet; RT: reverted diet (5 weeks of KD followed by 9 days of ND).

| | CT | KD | RT |
|---|---|---|---|
| PDH activity (mmol NADH/min/mg) | 90.97 ± 5.40 | 1.60 ± 0.12 | 36.80 ± 6.07 |
| p-PDH ser232 /E1 (relative to control) | 1.00 ± 0.56 | 33.39 ± 0.42 | 11.80 ± 1.58 |
| PDK4 / E1 (relative to control) | 1.00 ± 0.12 | 3.48 ± 0.37 | 0.87 ± 0.10 |
| CoASH (nmol/mg) | 0.618 ± 0.011 | 0.444 ± 0.069 | 0.585 ± 0.044 |
| Acetyl-CoA (nmol/mg) | 0.040 ± 0.011 | 0.244 ± 0.056 | 0.124 ± 0.055 |
| Acetyl-CoA/CoASH | 0.064 ± 0.019 | 0.550 ± 0.117 | 0.212 ± 0.093 |

## Supplementary Methods

*In Vivo MR Protocol*

Prior to *in vivo* imaging, each rat was anesthetized with 2 – 3 % isoflurane in 1.5 L/min oxygen, and the tail vein was cannulated with a catheter, which is connected to an extension line. Anesthetized rats were placed in a $^{13}$C/$^{1}$H dual-tuned birdcage RF coil (inner diameter = 80 mm) in the MRI scanner. The respiration and the body temperature were monitored throughout the experiment.

An integrated $^{1}$H/$^{13}$C MRI protocol (~20 minutes) was used. Following a three-plane localizer scan, spatial $B_0$ inhomogeneity around the heart was minimized using $^{1}$H point-resolved spectroscopy (PRESS) sequence by adjusting the shim currents. For $^{13}$C MRS, an axial slice (slice thickness = 10 mm) that included majority of the heart was prescribed, then was tilted to an oblique plane, if needed, to exclude other major organs such as liver. Acquisition of $^{13}$C MRS (spectral width = 5,000 Hz, #spectral points = 2,048) started with a bolus injection of 120-mM HP [1-$^{13}$C]pyruvate (1.5 mmol/kg body weight, injection rate = 0.25 mL/s, up to 4 mL) via the tail vein catheter and repeated every 3 seconds for 4 minutes. A 10$^{o}$ slice-selective RF pulse was used and the center frequency was set to [1-$^{13}$C]pyruvate by calculating from the water frequency of the $^{1}$H PRESS.[1]

*Tissue Preparation for NMR Isotopomer Analysis*

For the NMR metabolomic analysis, frozen heart tissue (~0.5 g) was extracted with 4 times sample size 5% perchloric acid and then lyophilized at -84°C using a FreeZone™ benchtop freeze dryer (Labconco Corp. Kansas City, MO, USA). Each sample was resuspended in 300 mL of $D_2O$ solution that contained 1-mM DSS and 1-mM ethylenediaminetetraacetate (EDTA). The $^{13}$C NMR spectra were acquired using a 14.1-T Avance III HD NMR spectrometer (Bruker, Billerica, MA, USA), equipped with a 10-mm cryoprobe (Bruker; spectral width = 36,057 Hz, acquisition time = 2 s, D1 = 6 s, #repetition = 8,000).

*Western Blot Analysis*

Anti-PDH-E1$\alpha$ (ab168379) and anti-phospho-PDH-E1$\alpha$ (pSer$^{232}$ AP1063) were purchased from Abcam and EMD Millipore, respectively. Rabbit polyclonal antisera to PDK4 was produced by Biosynthesis, Inc. to a mixture of following peptide sequences: CIPSREPKNLAKEKLA, DLVEFHEKSPEDQKALSE, and EFVDTLVKVRNRHHNVVPT linked to KLH. Specificity of antibody binding was validated by Western blot detection of protein at the appropriate molecular weight (Precision Plus Dual Color Standards, Bio-Rad, 1610374) and the requirement of primary antibody for secondary antibody binding. For analysis of mitochondrial proteins, mitochondria prepared in isolation buffer containing 20 mM NaF and 1 mM dichloroacetate were suspended in Laemmli sample buffer (Bio-Rad, 1610747) containing protease inhibitor cocktail (Sigma-Aldrich, P2714). Proteins (approximately 6 – 12 µg/lane, within the linear range) were resolved using 4 – 20% SDS-PAGE gradient gels (Bio-Rad, 4568096) and transferred to nitrocellulose membranes (0.2 µm). Membranes were incubated with primary antibodies at optimized dilution, washed, then incubated with fluorescent secondary antibodies (LI-COR, 925-32211). Proteins of interest were visualized and quantified using an Odyssey scanner and Image Studio Lite software (LI-COR). To reevaluate Western blots using a different antibody, membranes were stripped using NewBlot Nitro stripping buffer (LI-COR, 928-40030), checked for complete absence of prior signal, and then reprobed and reprocessed with appropriate primary and secondary antibodies.

*CoASH Analysis by HPLC-UV/Vis*

CoASH was extracted from snap frozen cardiac tissue crushed over liquid nitrogen. Approximately 30 – 50 mg of crushed frozen tissue was suspended in ice cold 0.35-M perchloric acid, 10-mM DTT by sonication. After 10 min on ice, proteins were pelleted by centrifugation at 16,000 $\times$ g and supernatants were filtered (0.45 µm PVDF). Protein pellets were resuspended in 150-mM KOH and protein content determined using the BCA assay. CoASH in the supernatants (100 µL, equivalent to approximately 0.3-0.5 mg protein) was resolved by ion-pair reverse phase HPLC (Waters XBridge C18 column, 150 $\times$ 4.5 mm$^2$, 5 µm) and detected by UV/Vis absorbance at 254 nm (Shimadzu LC-20AD HPLC system equipped with SIL-20A autosampler and photodiode array M20A UV/Vis detector). The mobile phase consisted of acetonitrile vs. 100 mM $KH_2PO_4$, 1 mM tetrabutylammonium sulfate, pH 6 using the following elution method with a flow rate of 1 mL/min: 0-10 min, 0-15% acetonitrile; 10-15 min, 15% acetonitrile; 15-17 min, 15-20% acetonitrile; 17-25 min, 20% acetonitrile;

25-30 min, 0% acetonitrile. CoASH levels were quantified using a standard curve constructed with known concentrations of CoASH (Sigma-Aldrich). Metabolite content is expressed as nmol/mg protein.
